# Ethyl 6-amino-5-cyano-2,4-bis­(4-methyl­phen­yl)-4*H*-pyran-3-carboxyl­ate

**DOI:** 10.1107/S1600536810035592

**Published:** 2010-09-11

**Authors:** M. Kannan, Kandhasamy Kumaravel, Gnanasambandam Vasuki, R. Krishna

**Affiliations:** a Centre for Bioinformatics, Pondicherry University, Puducherry 605 014, India; b Department of Chemistry, Pondicherry University, Puducherry 605 014, India

## Abstract

In the title compound, C_23_H_22_N_2_O_3_, the pyran ring adopts a twisted boat conformation. The tolyl rings and carboxyl­ate group are attached to the pyran ring with torsion angles of −77.1 (2), 59.5 (3) and 17.8 (3)°, respectively. The ethyl group is disordered over two orientations with a site-occupancy ratio of 0.508 (5):0.492 (5). In the crystal, mol­ecules are linked by N—H⋯N and N—H⋯O hydrogen bonds, generating a chain running the *a* axis. Weak C—H⋯O, C—H⋯N and C—H⋯π inter­actions are also observed.

## Related literature

For the use of related compounds in organic synthesis, see: Liang *et al.* (2009 ▶). For the synthesis, see: Vasuki & Kumaravel (2008 ▶). For conformational analysis, see: Cremer & Pople (1975 ▶). For related structures, see: Athimoolam *et al.* (2007 ▶); Kannan *et al.* (2010 ▶).
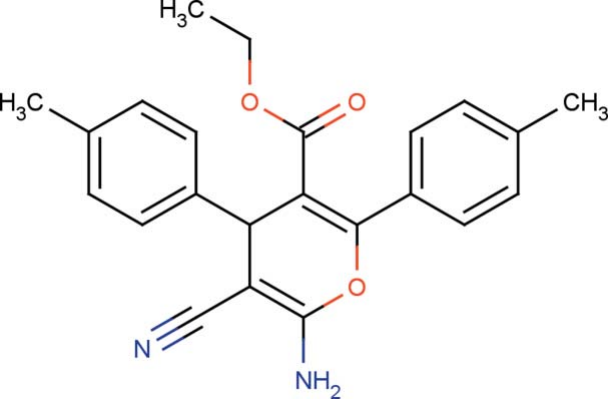


## Experimental

### 

#### Crystal data

C_23_H_22_N_2_O_3_
*M* *_r_* = 374.43
Triclinic, 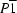

*a* = 8.3485 (2) Å
*b* = 11.4057 (3) Å
*c* = 11.6689 (3) Å
α = 72.902 (2)°
β = 72.690 (2)°
γ = 89.182 (2)°
*V* = 1010.64 (4) Å^3^
*Z* = 2
Mo *K*α radiation
μ = 0.08 mm^−1^
*T* = 293 K
0.30 × 0.20 × 0.20 mm

#### Data collection

Bruker Kappa APEXII CCD diffractometer
Absorption correction: multi-scan (*SADABS*; Bruker, 2004 ▶) *T*_min_ = 0.981, *T*_max_ = 0.984
18989 measured reflections
3554 independent reflections
2971 reflections with *I* > 2σ(*I*)
*R*_int_ = 0.022

#### Refinement

*R*[*F* ^2^ > 2σ(*F* ^2^)] = 0.066
*wR*(*F* ^2^) = 0.192
*S* = 1.04
3554 reflections
243 parameters
7 restraints
H-atom parameters constrained
Δρ_max_ = 0.56 e Å^−3^
Δρ_min_ = −0.58 e Å^−3^

### 

Data collection: *APEX2* (Bruker, 2004 ▶); cell refinement: *APEX2* and *SAINT* (Bruker, 2004 ▶); data reduction: *SAINT* and *XPREP* (Bruker, 2004 ▶); program(s) used to solve structure: *SHELXS97* (Sheldrick, 2008 ▶); program(s) used to refine structure: *SHELXL97* (Sheldrick, 2008 ▶); molecular graphics: *ORTEP-3 for Windows* (Farrugia, 1997 ▶); software used to prepare material for publication: *PLATON* (Spek, 2009 ▶).

## Supplementary Material

Crystal structure: contains datablocks I, global. DOI: 10.1107/S1600536810035592/ng5024sup1.cif

Structure factors: contains datablocks I. DOI: 10.1107/S1600536810035592/ng5024Isup2.hkl

Additional supplementary materials:  crystallographic information; 3D view; checkCIF report

## Figures and Tables

**Table 1:** Hydrogen-bond geometry (Å, °) *Cg* is the centroid of the C17–C22 ring.

*D*—H⋯*A*	*D*—H	H⋯*A*	*D*⋯*A*	*D*—H⋯*A*
N2—H2*A*⋯N1^i^	0.86	2.22	3.081 (3)	177
N2—H2*B*⋯O3^ii^	0.86	2.31	3.088 (3)	150
C6—H6⋯O3^iii^	0.93	2.51	3.432 (2)	171
C18—H18⋯N1^iv^	0.93	2.60	3.318 (3)	135
C23—H23*A*⋯*Cg*^v^	0.92	2.85	3.770 (4)	160

Symmetry codes: (i) 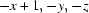; (ii) 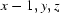; (iii) 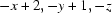; (iv) 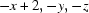; (v) 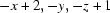.

**Fig. 1.**
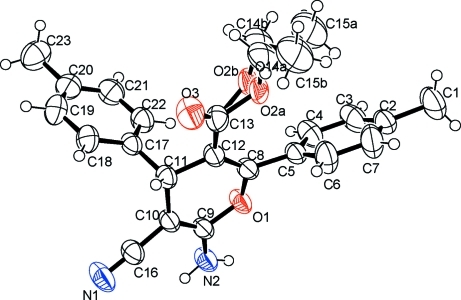
: The molecular structure of (I), showing the atom-numbering scheme and displacement ellipsoids drawn at the 50% probability level. The O-ethyl group is disordered over two positions with site-occupancy factor, from refinement of 0.508 (5) and 0.492 (5).

**Fig. 2.**
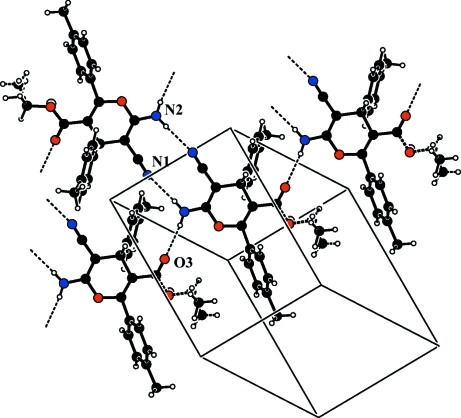
: The crystal packing of (I), showing intermolecular hydrogen bonding interactions as dashed lines.

**Fig. 3.**
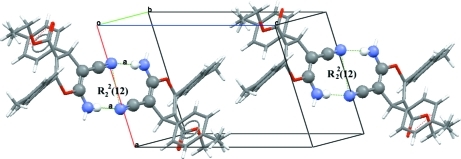
: A view of R22 (12) ring motifs formed by N—H···N interaction between to molecules and viewed along b* axis. The ring forming atoms are shown in ball and stick model and the Hydrogen bond are shown in green dashed lines.

**Fig. 4.**
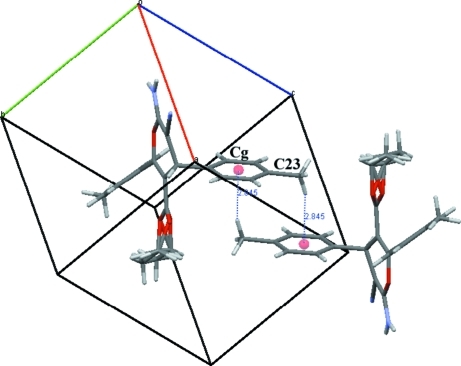
: The molecular interaction showing the weak C—H···pi interaction between two molecules (dashed line in blue color), In which the Cg is the centriod of C17—C22 ring. The disordered B section of the molecules omited for clarity.

## supplementary crystallographic information

### Comment

Like the title compounds some of the related compounds are widely used as organic intermediates in organic chemistry (Liang *et al.*, 2009). The title compound was synthesized by using Rapid four-component reactions in water (Vasuki & Kumaravel, 2008) and undertaken this study.

In the title compound (I), the pyran (A) ring adopts screw-boat conformation with the puckering parameters: Amplitude (Q) =0.221 (2) Å, Theta (θ) = 101.3 (5)° and Phi (φ) = 17.6 (7)°(Cremer & Pople, 1975), the puckered C_11_ atom having the maximum deviation of 0.137 (3) Å. The one toluene ring (Fig. 1) deviates significantly from planarity and attached with pyran ring by an (+)-*syn*-clinal conformation with torsion angle (C6—C5—C8—C12) of 59.5 (3)°, whereas the other toluene ring does not deviates from planarity and attached with pyran ring by torsion angle (C10—C11—C17—C22) of -77.1 (2)°, indiacting an (-)-*syn*-clinal conformtion. The carboxylate group is attached to pyran ring at C_12_ with the torsion angle of C_11_—C_12_—C_13_—O_3_ = 17.8 (3)°, indicating an (+)-*syn*-periplanar conformation. Moreover the *O*-ethyl group of carboxylate is disordered with the refined site-occupancy ratio of 0.508 (5)/0.492 (5). The two N—H···N and one N—H···O intermolecular interactions with a distance of 3.081 (4) Å and 3.088 (3) Å respectively are observed (Fig 2), in which the N—H···N intermolecular interaction forms *R*_2_^2^ (12) ring motifs. The weak C—H···O, C—H···N and C—H···π [C_g_: centroid of C_17_, C_18_, C_19_, C_20_, C_21_ and C_22_] (Fig. 3) intermolecular interaction also stabilized their three dimensional network of the crystal packing with a distance of 3.432 (3) Å, 3.318 (3) Å and 3.770 (4) Å respectively.

### Experimental

The title compound was prepared by the successive addition of 4-methylbenzaldehyde(0.240 g, 2 mmol) and piperidine (5 mol %) to a stirred aqueous mixture of malononitrile(0.132 g, 2 mmol) and ethyl 3-oxo-3-*p*-tolyl propanoate (0.412 g, 2 mmol) at room temperature under an open atmosphere with vigorous stirring for 5–10 min. The precipitated solid was filtered, washed with water and then with a mixture of ethylacetate/hexane (20:80) (Vasuki & Kumaravel, 2008). The product obtained was pure by TLC and ^1^H NMR spectroscopy. However, the products were further purified by recrystallization from ethanol. Analysis calculated for ethyl6-amino-5-cyano-2, 4-bis (4-methylphenyl)-4*H*-pyran-3-carboxylate showed that it has C_23_ H_22_ N_2_ O_3_.

### Refinement

The non-hydrogen atoms where refined anisotropically whereas hydrogen atoms were refined isotropically. The C—H and amine H atoms were positioned geometrically (C—H= 0.93–0.97 and N—H=0.86 Å) and were refined, using riding model with *U*_iso_(H) = xUeq(C), where *x* = 1.5 for methyl and 1.2 for all other atoms. The *O*-ethyl group of carboxylate shows disordered in two postition in the final refinement, the occupancy factors of two possible sites, O_2_B/C_14_B/C_15_B and O_2_A/C_14_A/C_15_A, converged to 0.508 (5) and 0.492 (5). For the disordered unit, the distance of C—C and C—O bond restrained to be 1.53 (10) Å and 1.43 (10) Å respectively.

### Crystal data

**Table:**

C_23_H_22_N_2_O_3_	*Z* = 2
*M**_r_* = 374.43	*F*(000) = 396
Triclinic, *P*1	*D*_x_ = 1.230 Mg m^−^^3^
Hall symbol: -P 1	Mo *K*α radiation, λ = 0.71073 Å
*a* = 8.3485 (2) Å	Cell parameters from 9717 reflections
*b* = 11.4057 (3) Å	θ = 2.2–32°
*c* = 11.6689 (3) Å	µ = 0.08 mm^−^^1^
α = 72.902 (2)°	*T* = 293 K
β = 72.690 (2)°	Rectangular, colourless
γ = 89.182 (2)°	0.30 × 0.20 × 0.20 mm
*V* = 1010.64 (4) Å^3^	

### Data collection

**Table:**

Bruker Kappa APEXII CCD diffractometer	3554 independent reflections
Radiation source: fine-focus sealed tube	2971 reflections with *I* > 2σ(*I*)
graphite	*R*_int_ = 0.022
Detector resolution: 0 pixels mm^-1^	θ_max_ = 25.0°, θ_min_ = 1.9°
ω and φ scan	*h* = −9→9
Absorption correction: multi-scan (*SADABS*; Bruker, 2004)	*k* = −13→13
*T*_min_ = 0.981, *T*_max_ = 0.984	*l* = −13→13
18989 measured reflections	

### Refinement

**Table:**

Refinement on *F*^2^	Secondary atom site location: difference Fourier map
Least-squares matrix: full	Hydrogen site location: inferred from neighbouring sites
*R*[*F*^2^ > 2σ(*F*^2^)] = 0.066	H-atom parameters constrained
*wR*(*F*^2^) = 0.192	*w* = 1/[σ^2^(*F*_o_^2^) + (0.0869*P*)^2^ + 0.7661*P*] where *P* = (*F*_o_^2^ + 2*F*_c_^2^)/3
*S* = 1.04	(Δ/σ)_max_ = 0.025
3554 reflections	Δρ_max_ = 0.56 e Å^−^^3^
243 parameters	Δρ_min_ = −0.58 e Å^−^^3^
7 restraints	Extinction correction: *SHELXL*
Primary atom site location: structure-invariant direct methods	Extinction coefficient: 0

### Special details

**Table:**

Geometry. All e.s.d.'s (except the e.s.d. in the dihedral angle between two l.s. planes) are estimated using the full covariance matrix. The cell e.s.d.'s are taken into account individually in the estimation of e.s.d.'s in distances, angles and torsion angles; correlations between e.s.d.'s in cell parameters are only used when they are defined by crystal symmetry. An approximate (isotropic) treatment of cell e.s.d.'s is used for estimating e.s.d.'s involving l.s. planes.
Refinement. Refinement of *F*^2^ against ALL reflections. The weighted *R*-factor *wR* and goodness of fit *S* are based on *F*^2^, conventional *R*-factors *R* are based on *F*, with *F* set to zero for negative *F*^2^. The threshold expression of *F*^2^ > σ(*F*^2^) is used only for calculating *R*-factors(gt) *etc*. and is not relevant to the choice of reflections for refinement. *R*-factors based on *F*^2^ are statistically about twice as large as those based on *F*, and *R*- factors based on ALL data will be even larger.

### Fractional atomic coordinates and isotropic or equivalent isotropic displacement parameters (Å^2^)

**Table:**

	*x*	*y*	*z*	*U*_iso_*/*U*_eq_	Occ. (<1)
C1	0.4425 (5)	0.8123 (3)	0.3388 (4)	0.0986 (13)	
H1A	0.3452	0.7959	0.4120	0.148*	
H1B	0.4206	0.8739	0.2701	0.148*	
H1C	0.5369	0.8412	0.3568	0.148*	
C2	0.4816 (3)	0.69499 (14)	0.30303 (19)	0.0648 (8)	
C3	0.4067 (2)	0.58140 (17)	0.38598 (15)	0.0720 (9)	
H3	0.3282	0.5769	0.4631	0.086*	
C4	0.4491 (2)	0.47452 (13)	0.35366 (15)	0.0618 (7)	
H4	0.3990	0.3985	0.4092	0.074*	
C5	0.5664 (2)	0.48123 (12)	0.23840 (15)	0.0473 (6)	
C6	0.6414 (2)	0.59482 (15)	0.15545 (14)	0.0718 (9)	
H6	0.7199	0.5993	0.0783	0.086*	
C7	0.5990 (3)	0.70170 (12)	0.18776 (18)	0.0803 (10)	
H7	0.6491	0.7777	0.1323	0.096*	
C8	0.6149 (3)	0.3670 (2)	0.2054 (2)	0.0449 (5)	
C9	0.4918 (3)	0.2162 (2)	0.1459 (2)	0.0466 (6)	
C10	0.6432 (3)	0.1734 (2)	0.1069 (2)	0.0472 (6)	
C11	0.7915 (3)	0.2049 (2)	0.1436 (2)	0.0453 (6)	
H11	0.8914	0.2207	0.0695	0.054*	
C12	0.7648 (3)	0.3221 (2)	0.1804 (2)	0.0453 (6)	
C13	0.9216 (3)	0.3763 (2)	0.1813 (2)	0.0532 (6)	
O2A	0.8984 (11)	0.4720 (4)	0.2391 (6)	0.0562 (13)	0.492 (5)
C14A	1.0588 (11)	0.5280 (9)	0.2238 (9)	0.072 (2)	0.492 (5)
H14A	1.1208	0.5657	0.1357	0.086*	0.492 (5)
H14B	1.1262	0.4692	0.2634	0.086*	0.492 (5)
C15A	1.0050 (13)	0.6246 (9)	0.2921 (12)	0.122 (3)	0.492 (5)
H15A	0.9331	0.6782	0.2539	0.184*	0.492 (5)
H15B	1.1029	0.6719	0.2862	0.184*	0.492 (5)
H15C	0.9453	0.5844	0.3791	0.184*	0.492 (5)
O2B	0.8938 (10)	0.4347 (5)	0.2773 (4)	0.0562 (13)	0.508 (5)
C14B	1.0311 (12)	0.5012 (7)	0.2866 (8)	0.072 (2)	0.508 (5)
H14C	1.1260	0.4507	0.2828	0.086*	0.508 (5)
H14D	0.9984	0.5159	0.3679	0.086*	0.508 (5)
C15B	1.0869 (14)	0.6244 (7)	0.1830 (9)	0.122 (3)	0.508 (5)
H15D	1.1080	0.6118	0.1022	0.184*	0.508 (5)
H15E	1.1879	0.6592	0.1877	0.184*	0.508 (5)
H15F	0.9997	0.6796	0.1941	0.184*	0.508 (5)
C16	0.6647 (3)	0.0898 (2)	0.0364 (3)	0.0536 (6)	
C17	0.82337 (19)	0.09928 (12)	0.24806 (13)	0.0442 (5)	
C18	0.95357 (19)	0.02588 (15)	0.21696 (12)	0.0555 (6)	
H18	1.0204	0.0420	0.1337	0.067*	
C19	0.9840 (2)	−0.07159 (15)	0.31037 (16)	0.0649 (7)	
H19	1.0711	−0.1207	0.2896	0.078*	
C20	0.8841 (2)	−0.09565 (14)	0.43487 (14)	0.0596 (7)	
C21	0.7539 (2)	−0.02225 (16)	0.46597 (11)	0.0626 (7)	
H21	0.6871	−0.0383	0.5493	0.075*	
C22	0.72354 (18)	0.07522 (15)	0.37256 (15)	0.0568 (7)	
H22	0.6364	0.1243	0.3934	0.068*	
C23	0.9202 (5)	−0.1997 (3)	0.5378 (3)	0.0826 (10)	
H23A	0.9932	−0.1682	0.5736	0.124*	
H23B	0.9736	−0.2618	0.5026	0.124*	
H23C	0.8165	−0.2349	0.6023	0.124*	
N1	0.6900 (3)	0.0234 (3)	−0.0223 (3)	0.0743 (8)	
N2	0.3450 (3)	0.1811 (2)	0.1388 (2)	0.0598 (6)	
H2A	0.3389	0.1238	0.1058	0.072*	
H2B	0.2560	0.2157	0.1672	0.072*	
O1	0.4731 (2)	0.30688 (16)	0.20301 (18)	0.0545 (5)	
O3	1.0577 (2)	0.35407 (19)	0.1320 (2)	0.0723 (6)	

### Atomic displacement parameters (Å^2^)

**Table:**

	*U*^11^	*U*^22^	*U*^33^	*U*^12^	*U*^13^	*U*^23^
C1	0.112 (3)	0.087 (2)	0.135 (3)	0.041 (2)	−0.050 (3)	−0.079 (3)
C2	0.0700 (18)	0.0665 (18)	0.086 (2)	0.0278 (14)	−0.0392 (16)	−0.0500 (16)
C3	0.0619 (17)	0.088 (2)	0.081 (2)	0.0153 (15)	−0.0124 (15)	−0.0570 (19)
C4	0.0554 (16)	0.0655 (17)	0.0693 (17)	0.0027 (13)	−0.0092 (13)	−0.0376 (14)
C5	0.0441 (12)	0.0527 (14)	0.0593 (14)	0.0138 (10)	−0.0217 (11)	−0.0324 (12)
C6	0.093 (2)	0.0535 (16)	0.0626 (17)	0.0186 (15)	−0.0089 (16)	−0.0245 (14)
C7	0.110 (3)	0.0493 (16)	0.080 (2)	0.0200 (16)	−0.0216 (19)	−0.0258 (15)
C8	0.0424 (12)	0.0490 (13)	0.0520 (13)	0.0063 (10)	−0.0163 (10)	−0.0264 (11)
C9	0.0454 (13)	0.0517 (13)	0.0560 (14)	0.0103 (10)	−0.0202 (11)	−0.0318 (11)
C10	0.0441 (13)	0.0514 (13)	0.0591 (14)	0.0115 (10)	−0.0191 (11)	−0.0329 (12)
C11	0.0371 (12)	0.0496 (13)	0.0564 (14)	0.0096 (10)	−0.0132 (10)	−0.0281 (11)
C12	0.0429 (13)	0.0448 (12)	0.0553 (14)	0.0074 (10)	−0.0161 (10)	−0.0249 (11)
C13	0.0471 (14)	0.0437 (13)	0.0726 (17)	0.0051 (10)	−0.0212 (12)	−0.0204 (12)
O2A	0.0596 (13)	0.029 (3)	0.092 (3)	0.007 (2)	−0.037 (3)	−0.022 (3)
C14A	0.067 (3)	0.061 (4)	0.100 (6)	−0.011 (3)	−0.031 (5)	−0.035 (5)
C15A	0.127 (7)	0.093 (4)	0.177 (8)	−0.009 (4)	−0.073 (6)	−0.058 (6)
O2B	0.0596 (13)	0.029 (3)	0.092 (3)	0.007 (2)	−0.037 (3)	−0.022 (3)
C14B	0.067 (3)	0.061 (4)	0.100 (6)	−0.011 (3)	−0.031 (5)	−0.035 (5)
C15B	0.127 (7)	0.093 (4)	0.177 (8)	−0.009 (4)	−0.073 (6)	−0.058 (6)
C16	0.0396 (13)	0.0638 (15)	0.0714 (17)	0.0123 (11)	−0.0192 (12)	−0.0396 (14)
C17	0.0354 (11)	0.0472 (12)	0.0590 (14)	0.0056 (9)	−0.0157 (10)	−0.0283 (11)
C18	0.0460 (14)	0.0573 (15)	0.0624 (16)	0.0134 (11)	−0.0101 (12)	−0.0238 (13)
C19	0.0545 (16)	0.0587 (16)	0.079 (2)	0.0183 (13)	−0.0179 (14)	−0.0213 (14)
C20	0.0549 (15)	0.0535 (15)	0.0720 (18)	−0.0024 (12)	−0.0233 (13)	−0.0170 (13)
C21	0.0607 (17)	0.0669 (17)	0.0587 (16)	0.0007 (13)	−0.0117 (13)	−0.0233 (14)
C22	0.0497 (14)	0.0629 (16)	0.0636 (16)	0.0121 (12)	−0.0136 (12)	−0.0318 (13)
C23	0.083 (2)	0.0685 (19)	0.089 (2)	−0.0005 (16)	−0.0342 (19)	−0.0051 (17)
N1	0.0520 (13)	0.0912 (18)	0.108 (2)	0.0174 (12)	−0.0227 (13)	−0.0750 (17)
N2	0.0434 (11)	0.0730 (15)	0.0879 (16)	0.0170 (10)	−0.0276 (11)	−0.0542 (13)
O1	0.0403 (9)	0.0644 (11)	0.0780 (12)	0.0137 (8)	−0.0196 (8)	−0.0489 (10)
O3	0.0395 (10)	0.0768 (13)	0.1065 (16)	0.0053 (9)	−0.0157 (10)	−0.0432 (12)

### Geometric parameters (Å, °)

**Table:**

C1—C2	1.518 (3)	C14A—C15A	1.5295 (10)
C1—H1A	0.9600	C14A—H14A	0.9700
C1—H1B	0.9600	C14A—H14B	0.9700
C1—H1C	0.9600	C15A—H15A	0.9600
C2—C3	1.3900	C15A—H15B	0.9600
C2—C7	1.3900	C15A—H15C	0.9600
C3—C4	1.3900	O2B—C14B	1.4309 (10)
C3—H3	0.9300	C14B—C15B	1.5295 (10)
C4—C5	1.3900	C14B—H14C	0.9700
C4—H4	0.9300	C14B—H14D	0.9700
C5—C6	1.3900	C15B—H15D	0.9600
C5—C8	1.482 (2)	C15B—H15E	0.9600
C6—C7	1.3900	C15B—H15F	0.9600
C6—H6	0.9300	C16—N1	1.140 (3)
C7—H7	0.9300	C17—C18	1.3900
C8—C12	1.329 (3)	C17—C22	1.3900
C8—O1	1.388 (3)	C18—C19	1.3900
C9—N2	1.328 (3)	C18—H18	0.9300
C9—C10	1.347 (3)	C19—C20	1.3900
C9—O1	1.371 (3)	C19—H19	0.9300
C10—C16	1.410 (3)	C20—C21	1.3900
C10—C11	1.505 (3)	C20—C23	1.520 (3)
C11—C12	1.514 (3)	C21—C22	1.3900
C11—C17	1.525 (3)	C21—H21	0.9300
C11—H11	0.9800	C22—H22	0.9300
C12—C13	1.460 (3)	C23—H23A	0.9600
C13—O3	1.172 (3)	C23—H23B	0.9600
C13—O2A	1.4256 (10)	C23—H23C	0.9600
C13—O2B	1.4261 (10)	N2—H2A	0.8600
O2A—C14A	1.4309 (10)	N2—H2B	0.8600
			
C2—C1—H1A	109.5	O2A—C14A—C15A	100.8 (5)
C2—C1—H1B	109.5	O2A—C14A—H14A	111.6
H1A—C1—H1B	109.5	C15A—C14A—H14A	111.6
C2—C1—H1C	109.5	O2A—C14A—H14B	111.6
H1A—C1—H1C	109.5	C15A—C14A—H14B	111.6
H1B—C1—H1C	109.5	H14A—C14A—H14B	109.4
C3—C2—C7	120.0	C13—O2B—C14B	119.8 (6)
C3—C2—C1	120.8 (2)	O2B—C14B—C15B	113.5 (6)
C7—C2—C1	119.1 (2)	O2B—C14B—H14C	108.9
C4—C3—C2	120.0	C15B—C14B—H14C	108.8
C4—C3—H3	120.0	O2B—C14B—H14D	108.9
C2—C3—H3	120.0	C15B—C14B—H14D	108.9
C3—C4—C5	120.0	H14C—C14B—H14D	107.7
C3—C4—H4	120.0	C14B—C15B—H15D	109.5
C5—C4—H4	120.0	C14B—C15B—H15E	109.5
C6—C5—C4	120.0	H15D—C15B—H15E	109.5
C6—C5—C8	120.01 (14)	C14B—C15B—H15F	109.5
C4—C5—C8	119.97 (14)	H15D—C15B—H15F	109.5
C5—C6—C7	120.0	H15E—C15B—H15F	109.5
C5—C6—H6	120.0	N1—C16—C10	176.8 (3)
C7—C6—H6	120.0	C18—C17—C22	120.0
C6—C7—C2	120.0	C18—C17—C11	118.98 (12)
C6—C7—H7	120.0	C22—C17—C11	121.02 (12)
C2—C7—H7	120.0	C17—C18—C19	120.0
C12—C8—O1	121.7 (2)	C17—C18—H18	120.0
C12—C8—C5	129.4 (2)	C19—C18—H18	120.0
O1—C8—C5	108.85 (17)	C20—C19—C18	120.0
N2—C9—C10	128.6 (2)	C20—C19—H19	120.0
N2—C9—O1	110.53 (19)	C18—C19—H19	120.0
C10—C9—O1	120.9 (2)	C19—C20—C21	120.0
C9—C10—C16	120.4 (2)	C19—C20—C23	120.16 (18)
C9—C10—C11	122.1 (2)	C21—C20—C23	119.81 (18)
C16—C10—C11	117.4 (2)	C20—C21—C22	120.0
C10—C11—C12	109.30 (18)	C20—C21—H21	120.0
C10—C11—C17	111.98 (19)	C22—C21—H21	120.0
C12—C11—C17	111.80 (18)	C21—C22—C17	120.0
C10—C11—H11	107.9	C21—C22—H22	120.0
C12—C11—H11	107.9	C17—C22—H22	120.0
C17—C11—H11	107.9	C20—C23—H23A	109.5
C8—C12—C13	126.6 (2)	C20—C23—H23B	109.5
C8—C12—C11	121.9 (2)	H23A—C23—H23B	109.5
C13—C12—C11	111.49 (18)	C20—C23—H23C	109.5
O3—C13—O2A	120.1 (4)	H23A—C23—H23C	109.5
O3—C13—O2B	120.3 (4)	H23B—C23—H23C	109.5
O2A—C13—O2B	20.6 (4)	C9—N2—H2A	120.0
O3—C13—C12	125.93 (19)	C9—N2—H2B	120.0
O2A—C13—C12	113.7 (4)	H2A—N2—H2B	120.0
O2B—C13—C12	111.8 (4)	C9—O1—C8	119.48 (17)
C13—O2A—C14A	109.7 (5)		
			
C7—C2—C3—C4	0.0	C8—C12—C13—O2A	13.2 (5)
C1—C2—C3—C4	−177.4 (2)	C11—C12—C13—O2A	−168.1 (3)
C2—C3—C4—C5	0.0	C8—C12—C13—O2B	35.5 (4)
C3—C4—C5—C6	0.0	C11—C12—C13—O2B	−145.8 (3)
C3—C4—C5—C8	178.43 (18)	O3—C13—O2A—C14A	0.6 (7)
C4—C5—C6—C7	0.0	O2B—C13—O2A—C14A	97.0 (19)
C8—C5—C6—C7	−178.43 (18)	C12—C13—O2A—C14A	−174.0 (5)
C5—C6—C7—C2	0.0	C13—O2A—C14A—C15A	−179.6 (8)
C3—C2—C7—C6	0.0	O3—C13—O2B—C14B	20.1 (7)
C1—C2—C7—C6	177.5 (2)	O2A—C13—O2B—C14B	−75.7 (19)
C6—C5—C8—C12	59.5 (3)	C12—C13—O2B—C14B	−175.3 (5)
C4—C5—C8—C12	−118.9 (3)	C13—O2B—C14B—C15B	73.4 (11)
C6—C5—C8—O1	−120.33 (18)	C9—C10—C16—N1	163 (6)
C4—C5—C8—O1	61.2 (2)	C11—C10—C16—N1	−21 (6)
N2—C9—C10—C16	7.7 (5)	C10—C11—C17—C18	102.33 (18)
O1—C9—C10—C16	−173.2 (2)	C12—C11—C17—C18	−134.63 (16)
N2—C9—C10—C11	−167.9 (3)	C10—C11—C17—C22	−77.07 (19)
O1—C9—C10—C11	11.2 (4)	C12—C11—C17—C22	46.0 (2)
C9—C10—C11—C12	−22.1 (3)	C22—C17—C18—C19	0.0
C16—C10—C11—C12	162.2 (2)	C11—C17—C18—C19	−179.41 (16)
C9—C10—C11—C17	102.4 (3)	C17—C18—C19—C20	0.0
C16—C10—C11—C17	−73.3 (3)	C18—C19—C20—C21	0.0
O1—C8—C12—C13	−179.3 (2)	C18—C19—C20—C23	−178.2 (2)
C5—C8—C12—C13	0.8 (4)	C19—C20—C21—C22	0.0
O1—C8—C12—C11	2.1 (4)	C23—C20—C21—C22	178.2 (2)
C5—C8—C12—C11	−177.8 (2)	C20—C21—C22—C17	0.0
C10—C11—C12—C8	15.4 (3)	C18—C17—C22—C21	0.0
C17—C11—C12—C8	−109.1 (3)	C11—C17—C22—C21	179.39 (16)
C10—C11—C12—C13	−163.4 (2)	N2—C9—O1—C8	−171.9 (2)
C17—C11—C12—C13	72.1 (2)	C10—C9—O1—C8	8.8 (4)
C8—C12—C13—O3	−160.9 (3)	C12—C8—O1—C9	−15.7 (4)
C11—C12—C13—O3	17.8 (4)	C5—C8—O1—C9	164.2 (2)

### Hydrogen-bond geometry (Å, °)

**Table:**

Cg is the centroid of the C17–C22 ring.

**Table:**

*D*—H···*A*	*D*—H	H···*A*	*D*···*A*	*D*—H···*A*
N2—H2A···N1^i^	0.86	2.22	3.081 (3)	177
N2—H2B···O3^ii^	0.86	2.31	3.088 (3)	150
C6—H6···O3^iii^	0.93	2.51	3.432 (2)	171
C18—H18···N1^iv^	0.93	2.60	3.318 (3)	135
C23—H23A···Cg^v^	0.92	2.85	3.770 (4)	160

Symmetry codes: (i) −*x*+1, −*y*, −*z*; (ii) *x*−1, *y*, *z*; (iii) −*x*+2, −*y*+1, −*z*; (iv) −*x*+2, −*y*, −*z*; (v) −*x*+2, −*y*, −*z*+1.
